# 

**Published:** 1914-08-15

**Authors:** 


### Medical Appointments.

St. Thomas's Hospital.—The following gentlemen have been selected as House Officers from Tuesday, August 4 and 18, 1914 (subject to the approval of the Treasurer): Casualty Officers and Resident Anæsthetists (to begin on August 18): R. M. de Mowbray, M.R.C.S., L.R.C.P.; V. C. Pennell, B.A. Cantab., M.R.C.S., L.R.C.P.; E. W. N. Hobhouse, M.A., M.B., B.Ch. Oxon., M.R.C.S., L.R.C.P.; L. G. Bourdillon, M.R.C.S., L.R.C.P.; E. L. K. Sargent, B.A., M.B., B.C. Cantab., M.R.C.S., L.R.C.P.; A. R. C. Doorly, M.R.C.S., L.R.C.P.; K. H. McMillan, M.R.C.S., L.R.C.P.; N. P. L. Lumb, M.R.C.S., L.R.C.P. Casualty Assistants (begun on August 4): F. L. Cassidi, B.A. Cantab., M.R.C.S., L.R.C.P.; W. H. Marshall, B.A. Cantab., M.R.C.S., L.R.C.P. Resident House Physicians (begun on August 4): J. A. G. Sparrow, M.A., M.B., B.Ch. Oxon.; G. T. Hebert, B.A., M.B., B.Ch. Oxon.; L. W. Shelley, B.A. Cantab., M.R.C.S., L.R.C.P.; J. C. Davies, B.A., M.B., B.Ch. Oxon., M.R.C.S., L.R.C.P. Resident House Surgeons (begun on August 4): G. A. Bird, M.B., B.S., B.Sc. Lond., M.R.C.S., L.R.C.P.; H. G. Chaplin, M.R.C.S., L.R.C.P.; C. W. Wheeler-Bennett, M.A., M.B., B.Ch. Oxon., M.R.C.S., L.R.C.P.; J. S. Sloper, M.B., B.S. Lond., M.R.C.S., L.R.C.P. House Surgeon to Block 8 (to begin on August 18): M. J. Petty, M.A., M.B., B.C. Cantab., F.R.C.S. Eng. Obstetric House Physicians (to begin on August 18): (Sen.) A. C. Ballance, M.A., M.B., B.Ch. Oxon., M.R.C.S., L.R.C.P.; (Jun.) H. P. Dawson, B.A., M.B., B.C. Cantab., M.R.C.S., L.R.C.P. Ophthalmic House Surgeons (to begin on August 18): (Sen.) C. L. Gimblett, B.A. Cantab., M.R.C.S., L.R.C.P.; (Jun.) P. G. Doyne, B.A., M.B., B.Ch. Oxon., M.R.C.S., L.R.C.P. Clinical Assistants (begun on August 4): Throat, J. E. Sharp, B.A. Cantab., M.R.C.S., L.R.C.P.; Skin, C. H. M. Gimlette, B.A. Cantab., M.R.C.S., L.R.C.P.; Ear, J. E. Sharp, B.A. Cantab., M.R.C.S., L.R.C.P.; Children's Surgical, C. W. Sparks, M.R.C.S., L.R.C.P.; Children's Medical, A. W. Dennis, B.A., M.B., B.Ch. Oxon., M.R.C.S., L.R.C.P.; A. F. Potter, M.R.C.S., L.R.C.P.

Rates of Subscription: Twelve months, 6/6; Six months, 4/-; Three months, 2/-, post free to any address in the United Kingdom. Abroad, Twelve months, 8/8; Six months, 5/-; Three months, 2/6, post free.—Address The Subscription Dept., "The Hospital," The Hospital Building, 28/29 Southampton Street Strand, London W.C.

